# Effects of Early Cumulus Cell Removal on Treatment Outcomes in Patients Undergoing *In Vitro* Fertilization: A Retrospective Cohort Study

**DOI:** 10.3389/fendo.2021.669507

**Published:** 2021-05-07

**Authors:** Pengcheng Kong, Mingru Yin, Chuanling Tang, Xiuxian Zhu, Orhan Bukulmez, Miaoxin Chen, Xiaoming Teng

**Affiliations:** ^1^ Centre for Assisted Reproduction, Shanghai First Maternity and Infant Hospital, Tongji University School of Medicine, Shanghai, China; ^2^ Department of Assisted Reproduction, Shanghai Ninth People’s Hospital, Shanghai Jiao Tong University School of Medicine, Shanghai, China; ^3^ Division of Reproductive Endocrinology and Infertility, Department of Obstetrics and Gynecology, University of Texas Southwestern Medical Center, Dallas, TX, United States

**Keywords:** short-term insemination, early cumulus cell removal, pregnancy outcomes, neonatal outcomes, *in vitro* fertilization

## Abstract

**Background:**

Early cumulus cell removal combined with early rescue intracytoplasmic sperm injection (ICSI) has been widely practiced in many *in vitro* fertilization (IVF) centers in China in order to avoid total fertilization failure. However, uncertainty remains whether the pregnancy and neonatal outcomes are associated with early cumulus cell removal.

**Objectives:**

To investigate if early cumulus cell removal alone after 4 hours co-incubation of gametes (4 h group), has detrimental effect on the pregnancy and neonatal outcomes in patients undergoing IVF, through a comparison with conventional cumulus cell removal after 20 hours of insemination (20 h group).

**Methods:**

This retrospective cohort study included 1784 patients who underwent their first fresh cleavage stage embryo transfer at the Centre for Assisted Reproduction of Shanghai First Maternity and Infant Hospital from June 2016 to December 2018 (4 h group, n=570; 20 h group, n=1214). A logistic regression analysis was performed to examine the independent association between early cumulus cell removal and pregnancy outcomes after adjustment for potential confounders. The neonatal outcomes between the two groups were compared.

**Results:**

When compared with the 20 h group, the 4 h group had similar pregnancy outcomes, including rates for biochemical pregnancy, clinical pregnancy, ongoing pregnancy, miscarriage, ectopic pregnancy, multiple pregnancy, live birth. There were 1073 infants delivered after embryo transfer (4 h group, n=337; 20 h group, n=736). Outcomes in both groups were similar for both singleton and twin gestations, including preterm birth rate and very preterm birth rate, mean birth weight, mean gestational age, sex ratio at birth and rate of congenital birth defects. In addition, findings pertaining to singleton gestations were also similar in the two groups for Z-scores (gestational age- and sex-adjusted birth weight), rates of small for gestational age, very small for gestational age, large for gestational age and very large for gestational age infants.

**Conclusions:**

In this study early cumulus cell removal alone was not associated with adverse pregnancy and neonatal outcomes. From this perspective, early cumulus cell removal to assess for a potential early rescue ICSI is therefore considered to be a safe option in patients undergoing IVF.

## Introduction

Total fertilization failure after conventional *in vitro* fertilization (IVF) is one of the most frustrating experiences in assisted reproductive technology (ART). Although IVF technology has advanced since 1978, the possibility of unexpected total fertilization failure is still unavoidable (1–4). Initially late-rescue intracytoplasmic sperm injection (ICSI) of 1-day-old unfertilized oocytes was used for total fertilization failure after conventional IVF, however aging oocytes contributed to poor clinical outcomes (5). Thus, short co-incubation of gametes, combined with early rescue ICSI, has been considered as an optimal strategy for avoiding total fertilization failure after conventional IVF (6, 7).

Notably, mammalian cumulus cells play a very important role during oocyte growth, maturation, fertilization and embryonic development (8, 9). In nature, cumulus cells are gradually shed from the oocyte after fertilization. However, in order to recognize unfertilized oocytes and also to perform early ICSI, early cumulus cell removal is required to establish the existence of a second polar body in oocytes 4 hours (4 h) after insemination (10). Thus, it remains unclear whether this early cumulus cell removal has any detrimental effects on the subsequent embryonic development after IVF. In fact, early cumulus cell removal may have both beneficial and adverse effects on oocytes and embryos. Since early cumulus cell removal reduces the co-incubation time between sperm and oocytes, it can reduce the levels of oxidative metabolites produced by cumulus cells and sperm, which may have detrimental effects on embryo developmental potential (11). Moreover, early cumulus cell removal reduces culture medium concentrations of estradiol and progesterone released by cumulus and corona cells. This concentration increases with the duration of incubation, which may impair embryo quality (12). However, it is harder to remove cumulus cells 4 h post-insemination compared with 20 hours (20 h) post-insemination, and the repeated aspiration may cause damage to cytoplasmic structures and subsequent embryonic development (13). Furthermore, early cumulus cell removal blocks inter-communication between cumulus cell and oocytes, which is essential for the formation of a competent oocyte. This may affect the development potential of the resultant embryos and the chance of successful pregnancies (14). Moreover, co-culture with autologous cumulus cells could enhance human embryo development and selection, implantation, and pregnancy rate in IVF cycles (9, 15, 16). Although several studies have investigated the effects of early cumulus cell removal on the embryonic and pregnancy outcomes of IVF, the results were inconclusive (13, 17, 18). In addition, few studies have examined the effects of early cumulus cell removal on live birth and neonatal outcomes.

In recent years, early cumulus cell removal alone or combined with early rescue ICSI has been widely practiced in many IVF laboratories in China. The aim of this study was to investigate the effects of early cumulus cell removal 4 h post-insemination on pregnancy and neonatal outcomes as compared to routine cumulus cell removal 20 h post-insemination in a retrospective cohort study.

## Materials and Methods

### Study Design and Patients

This was a retrospective, single-center cohort study in 1784 patients who underwent conventional IVF treatment at the Centre for Assisted Reproduction of Shanghai First Maternity and Infant Hospital from June 2016 to December 2018. The inclusion criteria were the following: Female less than 40 years of age; undergoing first IVF cycle with fresh cleavage stage embryo transfer; and having more than 4 oocytes retrieved. The exclusion criteria included early cumulus cell removal combined with early rescue ICSI cycles, use of donor eggs/sperm, women with congenital or secondary uterine abnormalities such as unicornuate uterus, septate uterus or uterine didelphys, adenomyosis, uterine submucosal fibroids, intrauterine adhesions, endometrial thickness < 7 mm on the day of embryo transfer, or severe oligospermia (total number of motile sperm <1 million after wash). This study was approved by the Research Ethics Committee of Shanghai First Maternity and Infant Hospital.

### Stimulation Protocols and Oocyte Retrieval

All patients received controlled ovarian stimulation (COS) treatment, which was performed by standard routines at the Centre. The COS treatment included gonadotrophin-releasing hormone agonist (GnRH-a) protocol, short GnRH-a protocol, gonadotrophin-releasing hormone antagonist (GnRH-ant) protocol or mild stimulation protocol as described previously (19). After two or more follicles reached a diameter of ≥18 mm, 10,000 IU of hCG (Lizhu, China) or 250 µg of hCG (Ovidrel; Italy) was injected subcutaneously to trigger final oocyte maturation. Oocyte retrieval was conducted 34-36 hours after hCG injection. The cumulus oocyte complexes (COCs) were collected in G-IVF PLUS medium (Vitrolife, Sweden) and incubated at 5% O_2_, 6% CO_2_, 37°C incubators for insemination.

### Sperm Preparation and Short-Term Insemination for Conventional IVF

Semen samples were collected by masturbation after 3 to 7 days of sexual abstinence on the day of oocyte retrieval. Semen analysis was performed according to the 2010 World Health Organization guidelines. The following sperm swim-up method was conducted. After 10-30 minutes of liquefaction in a 37°C incubator, 3 mL of G-IVF PLUS medium was gently stratified above the semen. The tube was inclined at a 45^°^ angle and was incubated for 1 hour (37°C, 6% CO_2_). The supernatant was then transferred into an empty tube and centrifuged for 5 minutes at 300 g. The sperm pellet was resuspended with warmed G-IVF Plus medium and maintained in a 6% CO_2_, 37°C incubator (Thermo Scientific, USA) until use. Three to four cumulus oocyte complexes (COCs) were placed into 100 µL of G-IVF PLUS droplets covered by mineral oil (Vitrolife, Sweden), and each oocyte was inseminated with 30,000 to 40,000 motile spermatozoa in a 5% O_2_, 6% CO_2_, 37°C incubator. A 4 h co-incubation of gametes was undertaken in all IVF cycles.

### Cumulus Cell Removal

Cumulus cells were mechanically removed after 4 h co-incubation of gametes (4 h group) from patients with unexplained infertility, primary infertility for more than three years, or where there was mild oligoasthenospermia (total motile sperm count ≥ 2 millions). The following method of early cumulus cell removal was used. Pasteur pipettes were pulled to become capillary pipettes over heat to achieve the diameters of approximately 150 µm, slightly larger than the oocyte. Oocytes were aspirated and blown out repeatedly until most of the cumulus cells were removed. The process excluded use of hyaluronidase. Care was exercised to prevent the damage on zona pellucida and oocytes. After most of the cumulus cells were removed, the zygotes in the 4 h group were then transferred to fresh G-1 plus (Vitrolife, Sweden) microdroplets. Fertilization was determined by the presence of two polar bodiesin a zygote after cumulus cells removal. Total fertilization failure was determined when none of the oocytes presented the second polar body. Less than 30% fertilization was classifies as a low fertilization rate. Patients with low fertilization rates or total fertilization failure were subjected to rescue ICSI at 6 h of insemination (20).

In contrast, conventional cumulus cell removal after 20 h of insemination (20 h group) was performed in patients without the indications of early cumulus cell removal. In this group, COCs were transferred from the insemination medium to fresh G-IVF PLUS microdroplets without sperm after 4 h co-incubation of gametes and cultured overnight. On day 1, the cumulus cells were removed at approximately 20 h of insemination to allow an assessment of pronuclear formation.

### Fertilization Assessment, Embryo Evaluation and Transfer

Oocytes of both groups were checked for the presence of two pronuclei (PN) to confirm fertilization approximately 20 h after oocyte insemination. Normal fertilization was determined when 2PN were present. Polyspermy was determined when ≥ 3PN were present. Embryos were graded by morphological assessment on day 2 or 3 after retrieval according to the standardized criteria (21). A maximum of two high-quality embryos was transferred on day 2 or 3 after retrieval under transabdominal ultrasound guidance. Patients received luteal support starting on the day of oocyte retrieval as described previously (19). In women with a positive hCG test, luteal-phase support was continued until 10 weeks gestation. All pregnant women were followed up for pregnancy outcomes until delivery or miscarriage.

### Outcome Measures

Biochemical pregnancy was defined as a positive pregnancy test result (serum hCG levels > 10 mIU/mL) 14 days after embryo transfer. Clinical pregnancy was defined as the presence of at least one gestational sac on ultrasound at 7 weeks. Ongoing pregnancy was defined as the presence of at least one fetus with heart motion on ultrasound beyond 12 weeks. Miscarriage rate was defined as the number of miscarriages before 28 weeks gestation divided by the number of women with positive pregnancy test. Multiple pregnancy was confirmed when more than one gestational sac was detected on ultrasound at 6 weeks. An infant born alive after 22 weeks of gestation was classified as a live birth. Preterm birth (PTB) and very PTB were defined as births that took place before 37 and 32 weeks gestation, respectively. Low birth weight (LBW) and fetal macrosomia were identified as birthweight <2500 g and >4000 g, respectively. Small for gestational age (SGA) and very SGA were identified as birthweight < 10th and < 3rd percentiles, respectively. Large for gestational age (LGA) and very LGA were identified as birthweight > 90th and >97th percentiles, respectively.

Additionally, the Z-score was calculated in accordance with the following equation: Z-score = (x - μ)/σ, in which x is the weight of a newborn, μ is the mean birthweight for infants in the same sex and same gestational age in the reference group, and σ is the standard deviation of the reference group. Birthweight percentiles and the calculation of Z scores were based on Chinese reference singleton newborns stratified by gestational age and sex at birth (22). The neonatal outcome data were obtained by telephone interview of the parents after delivery. The birth defects were classified and coded according to the International Classification of Diseases, 10th Revision (ICD-10).

### Statistical Analysis

Only the first transfer cycle outcomes of each included patient were analyzed. Quantitative variables were presented as mean ± standard deviation (SD) and compared by Student’s t test. Categorical variables were presented as % (n) and compared by the Chi-squared test or Fisher’s exact test as appropriate. Univariate analysis was performed to identify confounding variables that predicted pregnancy outcomes. Multivariate logistic regression analysis was performed to identify independent variables among potential confounding factors. Two criteria were used to select the covariates: 1) variables that were known as potential risk factors of IVF pregnancy outcomes based on current knowledge; 2) variables that were identified as significant in the univariate analysis. The results were reported as adjusted odds ratios (aORs) with 95% confidence intervals (CIs). Two-tailed P values <0.05 were considered significant. All statistical analyses were performed using the Statistical Program for Social Sciences (SPSS, Version 24.0, USA).

## Results

A total of 1784 cycles with fresh cleavage stage embryos transfer were analyzed in this study (**Table 1**). There were 570 cycles in the 4 h group and 1214 cycles in the 20 h group, respectively. The duration of infertility was significantly longer in the 4 h group than in the 20 h group (3.6 ± 2.2 *versus* 2.7 ± 2.0, *P*<0.001). There were also significant differences in causes of infertility between the two groups (*P*<0.001). The 4 h group had significantly higher total FSH dose, duration of stimulation, endometrial thickness and polyspermy rates compared with the 20 h group (*P*<0.05). There were no significant differences between the two groups in terms of age, BMI, basal FSH level, stimulation protocol, serum estradiol levels on the day of hCG administration, number of oocytes retrieved, number of embryos per transfer, rates of normal fertilization, high-quality embryos, and blastocyst rate.

**Table 1:** Demographic characteristics of patients.

	Early cumulus cell removal (4 h group)	Routine cumulus cell removal (20 h group)	p value
No. of patients	570	1214	
Female age (years)	32.6 ± 3.3	32.6 ± 3.4	0.974
Female BMI (kg/m^2^)	22.2 ± 3.2	22.0 ± 3.2	0.168
Duration of infertility (years)	3.6 ± 2.2	2.7 ± 2.0	**< 0.001**
Basal FSH level (IU/L)	6.3 ± 2.0	6.5 ± 2.0	0.115
Type of infertility %(n)			**< 0.001**
Primary	74.4(424/570)	45.4(551/1214)	
Secondary	25.6(146/570)	54.6(663/1214)	
Causes of infertility, %(n)			**< 0.001**
Female factor	52.1(297/570)	82.4(1000/1214)	
Male factor	17.9(102/570)	5.1(62/1214)	
Mixed	11.2(64/570)	7.5(91/1214)	
Unexplained	18.8(107/570)	5.0(61/1214)	
Stimulation protocol %(n)			0.442
Long agonist	82.1(468/570)	80.1(973/1214)	
Antagonist	15.4(88/570)	16.5(200/1214)	
Short agonist	1.8(10/570)	1.8(22/1214)	
Mild	0.7(4/570)	1.6(19/1214)	
Total FSH dosage (IU)	2299.5 ± 1214.9	2020.8 ± 874.1	**< 0.001**
Duration of stimulation (days)	11.6 ± 4.8	10.6 ± 2.9	**< 0.001**
Estradiol level on the hCG day (pg/ml)	2606.0 ± 1131.2	2638.5 ± 1160.0	0.648
Endometrial thickness (mm)	11.7 ± 2.4	11.3 ± 2.2	**0.002**
No. of oocytes retrieved	10.6 ± 3.8	10.6 ± 4.0	0.839
No. of embryos transferred	1.9 ± 0.4	1.9 ± 0.4	0.935
Stage of embryos transferred, %(n)			0.06
Day 2	2.8(16/570)	4.7(57/1214)	
Day 3	97.2(554/570)	95.3(1157/1214)	
Two pronuclei rate, % (n)	57.8(3502/6061)	59.2(7616/12860)	0.06
≥ Three pronuclei rate, % (n)	6.4(390/6061)	5.7(730/12860)	**0.040**
High-quality embryo rate, %(n)	40.6(1424/3502)	42.2(3215/7616)	0.128
Blastocyst rate, % (n)	39.4(637/1615)	38.7(1552/4006)	0.647

BMI, body mass index; FSH, follicle-stimulating hormone; hCG, human chorionic gonadotropin. Bold values mean statistically significant.

Pregnancy results are summarized in **Table 2**. After adjusting for female age, body mass index, duration of infertility, type of infertility, cause of infertility, duration of stimulation, total FSH dosage, normal fertilization rate, number of embryos transferred, stage of transferred embryos, and endometrial thickness, a logistic regression analysis was performed to explore the independent association between early cumulus cell removal and pregnancy outcomes. There were no significant differences in the rates of biochemical pregnancy, clinical pregnancy, ongoing pregnancy, live birth, miscarriage, ectopic pregnancy, multiple pregnancy and twin delivery between the two groups.

**Table 2:** Pregnancy outcomes of patients following fresh embryo transfers.

	Early cumulus cell removal (4 h group)	Routine cumulus cell removal (20 h group)	aOR [95% CI]	p value
No. of patients	570	1214		
Biochemical pregnancy rate, %(n)	56.3(321/570)	57.3(696/1214)	0.96[0.77,1.21]	0.74
Clinical pregnancy rate, %(n)	54.6(311/570)	54.9(666/1214)	0.96[0.77,1.20]	0.701
Ongoing pregnancy rate, %(n)	48.2(275/570)	48.5(589/1214)	0.95[0.76,1.18]	0.62
Miscarriage rate, %(n)	12.9(40/311)	11.9(79/666)	0.92[0.74,1.15]	0.463
Ectopic pregnancy rate, %(n)	2.9(9/311)	3.3(22/666)	0.86[0.37,1.97]	0.717
Multiple pregnancy rate, %(n)	17.0(97/1214)	19.1(232/1214)	0.87[0.65,1.17]	0.368
Live Birth Rate, %(n)	46.1(263/570)	47.4(575/1214)	1.08[0.70,1.66]	0.726
Twin delivery rate, %(n)	13.2(75/570)	13.9(169/1214)	0.92[0.66,1.28]	0.631

aOR, adjusted odds ratio; 95%CIs, 95% confidence intervals.

Regression model adjusted for female age, body mass index, duration of infertility, type of infertility, cause of infertility, duration of stimulation, total FSH dosage, normal fertilization rate, number of embryos transferred, stage of transferred embryos, and endometrial thickness.

In order to further evaluate the safety of early cumulus cell removal, neonatal outcomes between the 4 h and the 20 h group were analyzed (**Table 3**). A total of 1073 live born infants were included, and there were 337 and 736 newborns in the 4 h and the 20 h group respectively. No significant differences were found in mean birth weight and gestational age between the two groups, both in singleton and twin pregnancies. In addition, there were no significant differences between the two groups in the rates of preterm birth, very preterm birth, low birth weight, very low birth weight, fetal macrosomia, congenital malformation and sex ratio at birth, both in the singleton and twin pregnancies. Types of congenital malformations among live born infants between the two groups are shown in **Table 4**. Adjusted for newborn gender and gestational age, Z scores in the 4 h group (0.20 ± 0.98) were similar to those in the 20 h group (0.16 ± 0.99) in singleton pregnancy. In addition, no significant differences were observed in the rates of SGA, very SGA, LGA, very LGA infants between the two groups in singleton pregnancies.

**Table 3:** Neonatal outcomes of patients.

	Singletons		p value	Twins		p value
	Early cumulus cell removal (4 h group)	Routine cumulus cell removal (20 h group)		Early cumulus cell removal (4 h group)	Routine cumulus cell removal (20 h group)	
No. of patients	187	398		75	169	
Gestational age (weeks)	38.96 ± 1.33	38.96 ± 1.61	0.977	36.2 ± 1.8	36.3 ± 1.9	0.638
No. of preterm births (<37 weeks), %(n)	7.0(13/187)	5.5(22/398)	0.624	57.3(43/75)	50.9(86/169)	0.405
No. of very preterm births (<32 weeks), %(n)	0(0/187)	1.0(4/398)	0.402	2.7(2/75)	3.6(6/169)	0.975
Birth weight	3293.5 ± 447.3	3272.0 ± 487.8	0.611	2379.2 ± 438.7	2436.0 ± 423.7	0.178
Birth weight <1500 g, %(n)	0(0/187)	1.0(4/398)	0.402	4.7(7/150)	2.7(9/338)	0.275
Birth weight 1,500–2,499 g, %(n)	5.3(10/187)	4.3(17/398)	0.713	52.0(78/150)	46.4(157/338)	0.281
Birth weight 2,500–3,999 g, %(n)	87.7(164/187)	89.2(355/398)	0.694	43.3(65/150)	50.9(172/338)	0.141
Birth weight > 4,000 g, %(n)	7.5(14/187)	5.5(22/398)	0.462	0	0	
Neonatal malformation rate, %(n)	1.1(2/187)	2.8(11/398)	0.319	4.7(7/150)	6.2(21/338)	0.537
Newborn sex, %(n)						
Male, %(n)	52.4(98/187)	55.0(219/398)	0.594	54.7(82/150)	47.9(162/338)	0.202
Female, %(n)	47.6(89/187)	45.0(179/398)	0.594	45.3(68/150)	52.1(176/338)	0.202
Z-score	0.20 ± 0.98	0.16 ± 0.99	0.654			
Very small for gestational age (<3rd percentile), %(n)	3.2(6/187)	1.8(7/398)	0.419			
Small for gestational age (<10th percentile), %(n)	5.3(10/187)	7.3(29/398)	0.485			
Large for gestational age (>90th percentile), %(n)	12.3(23/187)	11.8(47/398)	0.973			
Very large for gestational age(>97th percentile), %(n)	5.9(11/187)	4.5(18/398)	0.615			

**Table 4:** Types of congenital malformations among live-born infants.

	Singletons		Twins	
	4 h group	20 h group	4 h group	20 h group
Any birth defect	2	11	7	21
Multiple defects	0	0	0	1
Congenital malformations of the nervous system (Q00–Q07)	0	1	0	1
Congenital malformations of eye, ear, face and neck (Q10–Q18)	0	1	0	1
Congenital malformations of the circulatory system (Q20–Q28)	1	3	3	11
Congenital malformations of the respiratory system (Q30–Q34)	0	1	1	1
Cleft lip and cleft palate (Q35–Q37)	0	1	0	1
Congenital malformations of the digestive system (Q38–Q45)	1	0	1	1
Congenital malformations of genital organs (Q50–Q56)	0	1	0	1
Congenital malformations of the urinary system (Q60–Q64)	0	1	1	1
Congenital malformations of the musculoskeletal system (Q65–Q79)	0	1	1	0
Chromosomal abnormalities, not elsewhere classified (Q90–Q99)	0	1	0	0
Other congenital malformations (Q80–Q89)	0	0	0	1
Metabolic abnormalities (E00–E90)	0	0	0	1

## Discussion

Once the sperm enters the oocyte, it immediately triggers calcium oscillation, further inducing cortical granular exocytosis. The released cortical granule proteins then induce zona pellucida reaction, blocking both the entry of other spermatozoa (23) and the bi-directional communication between the oocyte and the cumulus cells. In this large retrospective cohort study, no detrimental effects on pregnancy and neonatal outcomes in patients undergoing IVF treatment as a result of early cumulus cell removal 4 h after insemination could be demonstrated when compared with conventional cumulus cell removal 20 h after insemination. These findings suggested that once the mature oocytes were fertilized, the surrounding cumulus cells may not be essential for subsequent embryonic development.

In this study, no significant differences between the 4 h and 20 h groups were observed in the rates of biochemical pregnancy, clinical pregnancy, ongoing pregnancy, live birth, miscarriage, ectopic pregnancy, multiple pregnancy and twin delivery. Similarly, a small prospective randomized sibling-oocyte study involving 61 patients showed that 4 h group had no adverse influence on biochemical pregnancy and clinical pregnancy rates when compared with the 20 h group (13). Furthermore, another large sample retrospective study also indicated that early cumulus cell removal had no detrimental effects on clinical pregnancy, miscarriage and live birth rates when compared with conventional cumulus cell removal, in patients with high-risk of fertilization failure (24). Taken together, the data indicate that early cumulus cell removal may have no detrimental effects on pregnancy outcomes in fresh embryo transfer cycles.

There are always concerns about safety, when any type of modification is introduced into ART. Therefore, focusing on neonatal outcomes is well justified. The early cumulus cell removal is an important variable in conventional ART (25), and its potential effects need to be considered. Increasing evidence suggests that the birthweight is related to the risk of perinatal and infant morbidity and mortality, as well as to future adult chronic diseases (26, 27). Gestational age-specific birth weight is a commonly assessed perinatal outcome. Furthermore, fetal weight estimation using the customized birth weight percentiles has led to more accurate predictions of adverse perinatal outcomes (28). So far, studies on the neonatal outcomes of early cumulus cell removal are limited. This study found that both the singleton and twin pregnancies between 4 h and 20 h groups had similar neonatal outcomes such as birth weight, gestational age at delivery and preterm birth rate. In contrast, Guo et al. (29) showed that early cumulus cell removal had higher rates of low birth weight compared with conventional cumulus cell removal protocol. However, these results may be debated, because no distinction was made between singletons and twins, and the number of live births was small (n=54). The present study analyzed the neonatal outcomes in singleton and twin pregnancies separately, because twin pregnancies were associated with increased risk of adverse pregnancy and neonatal outcomes. This study also adjusted for newborn gender and gestational age in singleton pregnancies, and Z scores in two groups were also comparable. Thus, the data suggested that the early cumulus cell removal 4 h after insemination had no detrimental effects on neonatal outcomes in fresh embryo transfer cycles. However, follow-up studies are needed to examine the long-term effects of early cumulus cells removal on the offspring.

During the conventional IVF procedure, oocytes and cumulus cells were co-incubated for 19–20 h, and the cumulus cells were then removed to observe the fertilization status (29). It was known that cumulus cells provide oocytes with a series of factors which play important roles in nuclear and cytoplasmic maturation of oocytes, fertilization and development (8). Our data showed comparable rates for normal fertilization, high-quality embryos, blastocyst formation between the 4 h and the 20 h group, suggesting that normal fertilization and embryonic development were not affected by early cumulus cell removal. These results were consistent with the previous reports (13, 24).

Consistent with the previous reports (13, 29), our results showed a significantly higher polyspermy rate in the 4 h group as compared with the 20 h group. The oocytes may have been more vulnerable because of active spindles and microtubules shortly after insemination. It is possible that repeated aspirations for an earlier cumulus cell removal may have had adverse effects on the integrity of oocyte cytoplasmic structure, thus disrupting its defense mechanism against polyspermy, which may have resulted in additional sperm located by the cumulus cells or zona, gaining access to the oocyte (30). In addition, the potential temperature and pH fluctuations during the process of early cumulus cell removal and observation may have also played a role. In contrast, several studies have shown that early cumulus cell removal does not increase the polyspermy rate (17, 30). This discrepancy may be due to the different degree or time of cumulus cell removal in different studies. Nevertheless, in this study, the increased polyspermy rate with early cumulus removal did not affect the major ART outcomes. Therefore, the clinical significance of this finding may be limited.

In China, the indication and proportion of ICSI cycles per center is strictly regulated by the government (7). Therefore, early cumulus cell removal was applied for patients with a higher risk of fertilization failure to avoid the excessive use of ICSI technique in clinical practice. Previous studies suggested that primary infertility and longer infertility duration were important risk factors for total fertilization failure (31, 32). The incidence of total fertilization failure was also higher in patients with unexplained infertility (33, 34). Patients with these causes of infertility were included in the 4 h group, and early rescue ICSI was performed if necessary, in this study. This can account for the significant difference between the 4 h and the 20 h groups for rates of primary infertility, duration of infertility, type of infertility and causes of infertility. Compared with the 20h group, the 4 h group had significantly higher total FSH dose, duration of stimulation, and endometrial thickness. This may be due to suboptimal ovarian response, which is associated with the different characteristics of patients in the 4 h group.The major strength of the current study was to focus on safety aspects of early cumulus cell removal with a large sample size. In addition, laboratory practices were consistent during the study period, to minimize possible confounders associated with pregnancy and neonatal outcomes. There are some limitations to this study. As a retrospective design, data were collected from medical records, which could not provide all information on personal covariates. Limitations of this retrospective study were minimized by adjusting for the known factors related to the IVF outcomes in the multivariable analysis as independent variables. In particular, the information on congenital malformations was obtained by parental report after delivery and, the data on patients with miscarriage outcome were not available. The rate of congenital malformations was calculated using live newborns, and therefore the data did not represent all birth defects. Another limitation was the selection of patient population and the different sample size of the two study groups. Therefore, prospective multicenter trials in general IVF population are needed to eliminate the effects of the confounders.

## Conclusions

In conclusion, results from this study suggest that early cumulus cell removal after 4 h co-incubation of gametes has no apparent effects on pregnancy and neonatal outcomes when compared with conventional cumulus cell removal, during fresh cleavage stage embryo transfer cycles. Thus, early cumulus cell removal to assess for a potential early rescue ICSI seems to be safe in terms of pregnancy and live birth outcomes, thereby reducing the utilization rate of ICSI in assisted reproduction treatment. However, the long-term follow-up studies of the children conceived through early cumulus cell removal are still needed to further validate the safety of early cumulus cell removal.

## Data Availability Statement

The original contributions presented in the study are included in the article/supplementary material. Further inquiries can be directed to the corresponding authors.

## Ethics Statement

This study was approved by the medical ethics committees of the Shanghai First Maternity and Infant Hospital, Tongji University School of Medicine and informed consent was obtained from all participants.

## Author Contributions

XT and MC conceived and designed this study. PK, MY, CT, and XZ contributed to data acquisition, analysis and interpretation and drafted the manuscript. MC and OB were involved in the study critical discussion and revision of the manuscript. All authors contributed to the article and approved the submitted version.

## Funding

This study was supported by five grants from the National Natural Science Foundation of China (81801538, 81871213, 81671468, 81701523, 81971383), a grant from the State’s Key Project of Research and Development Plan (SQ2017ZY050118-03), a grant from the Special Funds for Clinical Medical Research of Chinese Medical Association (18010030732), a grant from the Clinical Research Plan of Shanghai Hospital Development Center (SHDC2020CR4080), two grants from the Science and Technology Commission of Shanghai Municipality (19411960500, 19411960600), and a grant from the Natural Science Foundation of Shanghai (17ZR1422000). The funding bodies have not participated in the design of the study and collection, analysis, interpretation of data or in writing the manuscript.

## Conflict of Interest

The authors declare that the research was conducted in the absence of any commercial or financial relationships that could be construed as a potential conflict of interest.
